# Foliar application of specific yeast derivative enhances anthocyanins accumulation and gene expression in Sangiovese cv (*Vitis vinifera* L.)

**DOI:** 10.1038/s41598-020-68479-0

**Published:** 2020-07-15

**Authors:** C. Pastore, G. Allegro, G. Valentini, A. Pizziolo, F. Battista, F. Spinelli, I. Filippetti

**Affiliations:** 1 Department of Agricultural and Food Sciences, University of Bologna, Viale Fanin, 44, 40127 Bologna, Italy; 2 Lallemand Inc. Italy, Via Rossini 14/B, 37060 Castel d’Azzano, Verona Italy

**Keywords:** Plant sciences, Plant molecular biology, Plant physiology, Secondary metabolism

## Abstract

The effect of elicitors on secondary metabolism in vines is receiving much interest, since it has been shown that they are able to increase the accumulation of phenolics, especially anthocyanins. This research aims to investigate the biochemical and molecular effects of the application of a commercial yeast derivative (*Saccharomyces cerevisiae*) on the accumulation of anthocyanins in potted Sangiovese vines. Experiments were performed on three consecutive years and the yeast derivative was applied at the beginning and at the end of veraison. Technological ripening, accumulation of anthocyanins and expression of the main genes involved in their biosynthesis were assessed. Technological ripening proceeded in a similar way in both treated and untreated berries in the three years. A significant increase in the concentration of anthocyanins was instead detected, following the induction by the yeast derivative of the expression of the genes involved in their biosynthesis. The research highlights the possibility of applying a specific inactivated yeast to increase the anthocyanin concentration even under the current climate change conditions, in Sangiovese, a cultivar extremely sensitive to high temperatures.

## Introduction

In premium red wine production, the berry colour is a key factor and during the last years, due to climate change, achieving an optimal level of berry and wine colour has been a hard challenge for growers^1,2^. Indeed, global warming accelerates berry ripening processes, that, especially in red varieties, can result in unbalanced wines, with too high alcoholic content, low acidity and poor colour^3–6^, also due to the accumulation of brown compounds induced by sunburn damages in the skins of the berries directly exposed to excessive sunlight and high temperatures^7^. In addition, in the current context of climate change, the early achievement of the optimal grape technological ripening with the right balance between sugar content and acidity, could lead to uncompleted phenolic maturity involving also seed tannins that, in this condition, could elicit astringent and bitter sensation in wine^8^.

In recent years, several agronomic practices applied during the vegetative season have been developed to counteract this situation, such as post-veraison trimming^5,9^ or defoliation of the apical part of the shoots^10^, with the purpose of slowing down the accumulation of soluble solids, delaying harvest and favouring the accumulation of anthocyanins, also increasing their extractability^3^. Natural elicitors based on microbial or plant extract are an interesting strategy to increase secondary metabolite accumulation^11^. They can be classified by their type as abiotic elicitors (inorganic salts, and physical factors acting as elicitors like Cu and Cd ions, Ca^2+^ and treatments inducing high cellular pH) or biotic elicitors that include polysaccharides derived from plant cell walls (pectin or cellulose), microorganisms (chitin or glucans) and glycoproteins or G-protein or intracellular proteins^12^. Elicitors were first used to increase plant resistance to pathogens, although it was later found that the mechanism involved may also increase polyphenol levels^13–15^. Their use on plants aims to trigger a response leading to secondary metabolites synthesis such as phytoalexins, stilbenes, anthocyanins and tannins through the activity of recognition on plasma membrane receptors^14–17^ studied from long time. The receptors involved in the perception of elicitors in plants are mainly still unknown and only recently, Brulè et al.^18^ described one receptor kinases with activity involved in inducing immune responses in *Vitis vinifera* Cabernet Sauvignon, following chitosan treatment.

In grapevine, the application of elicitors such as chitosan, was able to induce phenylalanine ammonia lyase (PAL) activity on both berries^19^ and leaves^20^. Also, pectin-derived oligosaccharides (PDOs) could enhance PAL activity^21^ and increase anthocyanin concentrations in Flame Seedless^22^ and Cabernet Sauvignon, when applied on clusters in pre-veraison^23^.

Inactivated yeast, mostly derived from cultured *Saccharomyces cerevisiae* strains that are then subjected to thermal or enzymatic inactivation, are considered biotic elicitors as they are composed of mannoproteins, β-1,3- and β-1,6-glucans and chitin, lipids, sterols, and proteins^24^. It has been reported that the yeast derivative application triggers plant defence mechanisms, leading to an accumulation of secondary metabolites, such as phenols, sesquiterpenoids, and other aromatic compounds both in several plant cultures^25–29^ and in grapevine in field conditions^30–33^. In particular, in the Tempranillo red berry grape variety, foliar treatments with a specific inactivated yeast increased grape and wine anthocyanin contents when compared to the control^32^.

As it is well-known, genetic control plays a major role in the synthesis and accumulation of anthocyanin compounds in a given cultivar, but different genotypes may show a dissimilar response in terms of anthocyanins due to interaction with the climatic conditions^34^, the vintages and the cultivation sites^35^, and the application of different cultural practices^36–38^. In Sangiovese, the most widely cultivated Italian variety, the influence of environmental conditions during ripening on anthocyanin accumulation and profile is particularly strong and Sangiovese has been shown to be very sensitive to air temperature increase in the range of 26–35 °C^39,40^.

Given that the effect of yeast derivatives on the expression of structural and regulatory genes of the phenylpropanoid pathway has not been evaluated yet, this study aims to assess the effectiveness of a commercial yeast derivative on *V. vinifera* cv. Sangiovese berry skin anthocyanin accumulation and changes in expression of structural and regulatory genes of the anthocyanin pathways.

## Results

### Monitoring of technological ripening and yield parameters at harvest

**Figure 1:**
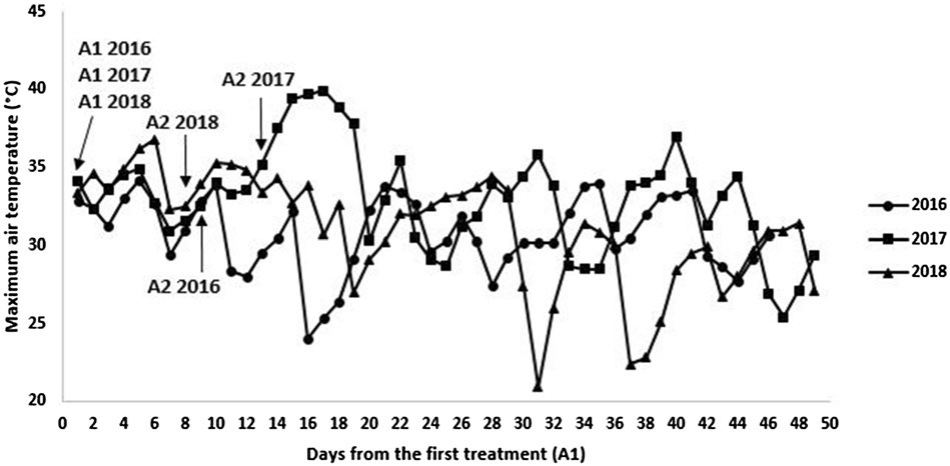
Trends in maximum air temperature from the first application of yeast derivative (A1) to harvest in 2016, 2017 and 2018 seasons. Arrows indicate the date on which first (A1) and second (A2) treatments were applied for each year.

Temperatures during the vine vegetative period were very different in the three years. Degree Days (DD), calculated according to Amerine and Winkler^41^, resulted 1,935, 2,062 and 2,213 DD in 2016, 2017 and 2018, respectively. Considering the monthly average temperature, 2016 was the coldest year, differing especially in August, with average temperatures 2.6 and 2.2 °C lower than 2017 and 2018, respectively (Table 1). Although 2018 was the warmest year in terms of DD in the period April-September, the average temperature in August did not differ from year 2017 (Table 1). In 2017, in the week after the second application of the specific inactivated yeast (July, 31, 12 days after the first treatment), an intense heat wave occurred being maximum temperature 40 °C for several consecutive days (Fig. 1).

**Table 1:** April to September average monthly temperatures (°C) and total rainfall (mm) in the three seasons (2016, 2017 and 2018) in which the experiment was conducted.

	Average T (°C)			Total rainfall (mm)		
	2016	2017	2018	2016	2017	2018
April	14.6	14.1	16.0	45	33.4	65
May	17.3	18.4	19.6	72.4	55.8	78.3
June	21.7	24.5	23.4	107.0	21	63.6
July	25.5	25.8	25.9	27.8	8	42.8
August	23.7	26.3	25.9	31.8	25.2	33.5
September	21.1	18.3	21.6	33.2	117.8	36

Total rainfall was lower in 2017 with a total of 261 mm in the April-September period. In the same period, rainfall was 317 and 319 mm in 2016 and 2018, respectively.

In each year, the yeast derivative treatment did not result neither during ripening nor at harvest in significant changes in the accumulation of soluble solid content (°Brix, Table 2, Supplementary Fig. 1), titratable acidity (Table 2, Supplementary Fig. 2) and pH (Table 2) in comparison to untreated vines. Despite no year × treatment significant interactions were detected in technological ripening compounds, significant differences among years were detected for sugar accumulation, being the 2018 the year in which the lowest sugar concentrations both in C and LVM berries were reached (Table 2, Supplementary Fig. 1).

**Table 2:** Total soluble solids (°Brix), titratable acidity (TA) and pH in control (C) and yeast derivative (LVM) treated berries at harvest. Data averaged over 2016–2018.

	°Brix	TA (g/L)	pH
C	21.9	6.8	3.35
LVM	22.3	6.3	3.34
Sign	ns	ns	ns
Year effect	*	ns	ns
Treatment × year interaction	ns	ns	ns

Means within columns followed by different letters differ significantly, as calculated by Tukey statistical analysis (P < 0.05). Asterisks indicate significance at P < 0.05.

*ns* not significant.

Yield was not influenced by the treatment in any of the three years as well as the number of clusters and their weight. The average of three years cluster number per vine was uniform in both treatments at around 15, while cluster weight was in the average 95 g.

## Anthocyanin accumulation and composition during ripening

**Figure 2:**
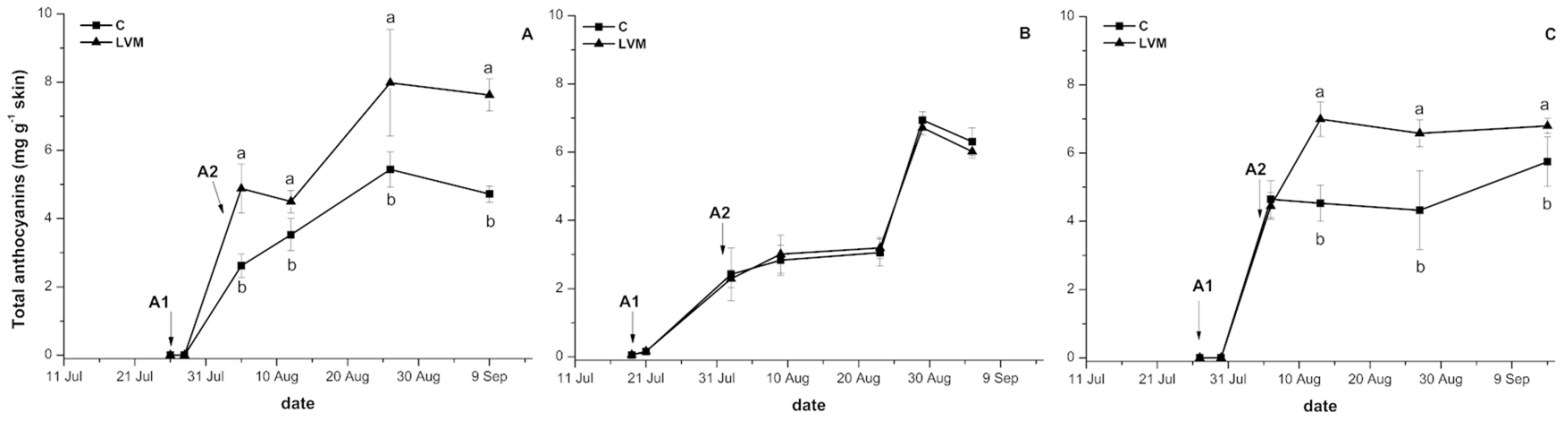
Anthocyanin accumulation in control (C) and yeast derivative (LVM) treated berries during 2016 (**A**), 2017 (**B**) and 2018 (**C**) seasons. Arrows indicate the date on which first (A1) and second (A2) treatments were applied for each year. Error bars indicate standard error (*n* = 3). At each sampling date means followed by different letters differ significantly, as calculated by Tukey statistical analysis (P < 0.05).

In our experimental condition, the effectiveness of the treatment on anthocyanin accumulation was affected by the weather conditions of each season and both statistically significant treatment*year interaction at harvest and different behaviour following the treatment, were detected among years for C and LVM berries (Fig. 2). Differently from what observed for technological ripening, no year effect was instead recorded.

In all years, since the analyses of anthocyanin were performed starting with the beginning of veraison, an initial linear increase in anthocyanin concentration was observed in both C and LVM berries. However, anthocyanin concentration was significantly higher in LVM treated berries, both in 2016 and 2018, and the differences were maintained until harvest (Fig. 2A,C). In 2017, instead, there was no influence of the treatment on anthocyanin evolution, although a very slight increase was observed following the second application (Fig. 2B).

The yeast derivative application did not entail any modification in the anthocyanin composition of berries at harvest which, in the three years, preserved the typical anthocyanin profile of Sangiovese (Supplementary Table 1), characterized almost exclusively by the glucosylated form of the five main anthocyanin compounds present in grapevine (delphinidin, cyanidin, petunidin, peonidin and malvidin) and with acylated forms derivatives accounting for no more than 2% of total anthocyanins, as previously reported^42^.

### Expression analysis on the genes involved in the biosynthesis of anthocyanin in berries

**Figure 3:**
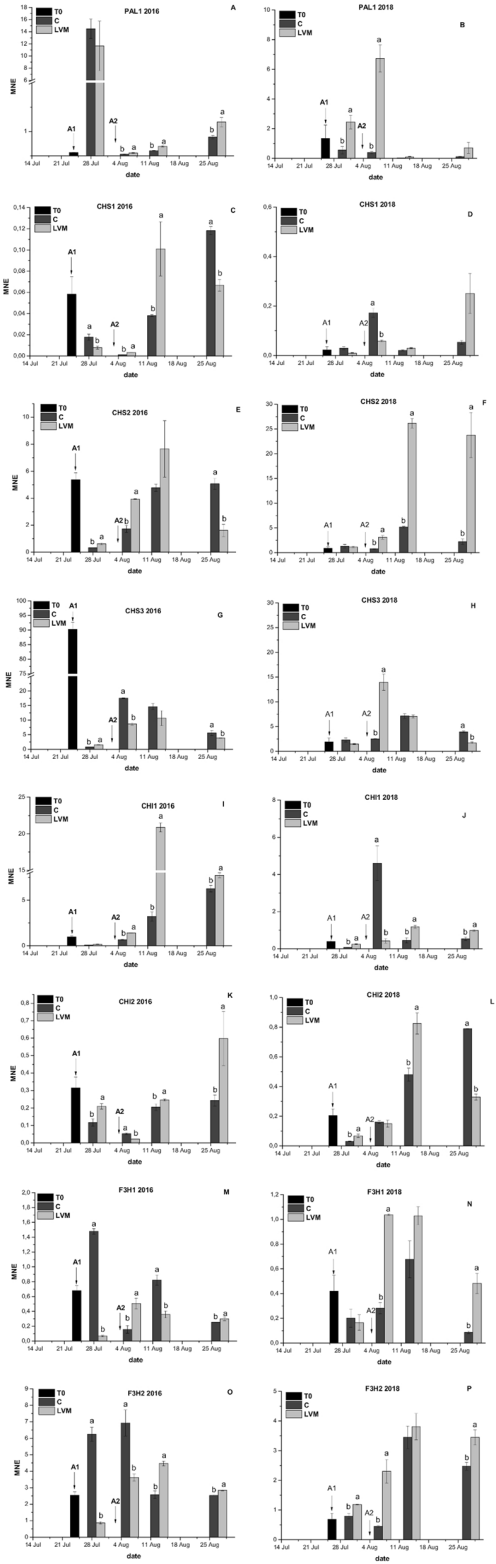
Expression analyses of the genes involved in the early step of flavonoid biosynthesis on 2016 and 2018 samples in control (C) and yeast derivative (LVM) treated berry skin: PAL1 (phenylalanine ammonia lyase; **A**, **B**); CHS1 (chalcone synthase 1; **C**, **D**) CHS2 (chalcone synthase 2; **E**, **F**) CHS3 (chalcone synthase 3; **G**, **H**), CHI1 (chalcone isomerase 1; **I**, **J**), CHI2 (chalcone isomerase 2; **K**, **L**), F3H1 (flavanone 3-hydroxylase 1; **M**, **N**) and F3H2 (flavanone 3-hydroxylase 2; **O**, **P**). Arrows indicate the date on which first (A1) and second (A2) treatments were applied for each year. T0 analyses were conducted on three different samples, each deriving by the combination of C and LVM berries. Error bars indicate standard error (n = 3). At each sampling date means followed by different letters differ significantly, as calculated by Tukey statistical analysis (P < 0.05).

**Figure 4:**
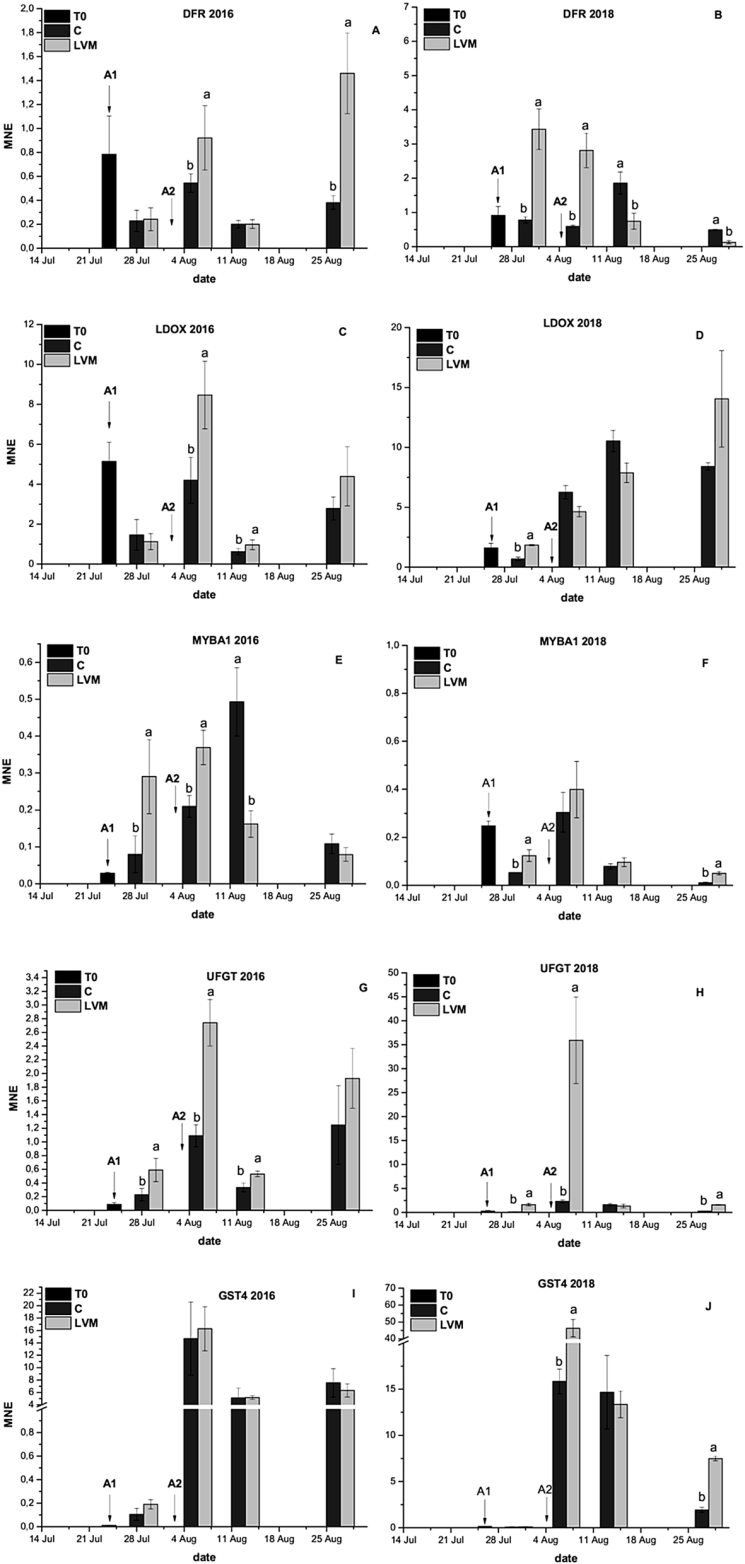
Expression analyses of genes involved in the late steps of anthocyanin biosynthesis and in their regulation and transport on 2016 and 2018 samples in control (C) and yeast derivative (LVM) treated berry skin: DFR (dihydroflavonol reductase; **A**, **B**), LDOX (leucoanthocyanidin dioxygenase; **C**, **D**), UFGT (UDP-glucose:flavonoid 3-O-glucosyl transferase; **E**, **F**), MYBA1 (**G**, **H**), GST4 (glutathione S-transferase; **I**, **J**). Arrows indicate the date on which first (A1) and second (A2) treatments were applied for each year. T0 analyses were conducted on three different samples, each deriving by the combination of C and LVM berries. Error bars indicate standard error (n = 3). At each sampling date means followed by different letters differ significantly, as calculated by Tukey statistical analysis (P < 0.05).

Gene expression analyses were performed on C and LVM berry skins in 2016, 2017 and 2018 on all genes involved in the biosynthesis of the early (PAL1, CHS1, CHS2, CHS3, CHI1, CHI2, F3H1 and F3H2, Fig. 3, Supplementary Fig. 3) and late (DFR, LDOX, UFGT and MYBA1, Fig. 4, Supplementary Fig. 4) steps of anthocyanin biosynthesis and on one gene involved in anthocyanin transport to the vacuole^43^ (GST4, Fig. 4, Supplementary Fig. 4).

As a general rule, a correlation was found between the effectiveness of the treatment on anthocyanin accumulation and the gene expression analyses, as differences between C and LVM berries in terms of gene expression were detected in 2016 and 2018, while in 2017 (Supplementary Figs. 3, 4) no differences were recorded at any sampling date.

Concerning the genes involved in the early steps of flavonoid biosynthesis a general increase of their expression in LVM berries was detected in 2016 and 2018 (Fig. 3). In particular, CHI2 and F3H1 showed a constant behaviour in both years and an induction of expression following the first and second application of the specific inactivated yeast was recorded (Fig. 3K–N). For the other genes, differences in the timing of activation were detected among seasons. In the case of PAL1, CHI1 and F3H2 a prompter induction was detected in 2018 (Fig. 3B,J,P) than in 2016 (Fig. 3A,I,O) and a peak of expression was recorded in LVM berries immediately after the first treatment in 2018, while in 2016, these genes were overexpressed in comparison to C after the second treatment or even later. An opposite behaviour was observed for CHS3, whose expression was instead activated earlier in LVM berries in 2016 (Fig. 3G) than in 2018 (Fig. 3H).

Among the genes involved in the specific steps of anthocyanin biosynthesis (Fig. 4 and Supplementary Fig. 4), in both years a prompter induction in LVM berries was observed in comparison to C, immediately following the first treatment, for UFGT (Fig. 4F,H) and its transcriptional regulation MYBA1 (Fig. 4E,F). A similar behaviour was recorded for DFR and LDOX in 2018 (Fig. 4B,D), while in 2016 their expression peaked only after the second application (Fig. 4A,C).

A less clear effect of the treatment was observed for the expression of GST4, which was stimulated only in 2018 (Fig. 4I,J).

## Discussion

In this research, we evaluated a new strategy that may be useful to increase anthocyanin concentration in Sangiovese cultivar without interfering with sugar accumulation. The approach is particularly interesting in the current context of climate change, where increasing temperatures often involve a decoupling of sugars and anthocyanin accumulation that results in a too much rapid accumulation of sugars in comparison to anthocyanins and negatively affects berry and wine composition. Due to the different nature of elicitors it is difficult to univocally predict their specific effect. In recent studies, it was reported that the biostimulation through the application of a seaweed extract (Ascophyllum *nodosum*) in Tempranillo grapevines did not affect neither berry ripening nor colour intensity of the derived wines, but it had strong impact on amino acid content in must^44^. In similar experiments conducted on Merlot, the greatest vine responses were obtained with 1% extract concentration and N supply and involved no changes in berry ripening, but an increase in anthocyanin extractability of berry skins^45^. The application of different elicitors can instead affect the course of berry ripening, contemporarily influencing berry sugar and anthocyanin accumulation, and recently both sugar and anthocyanin concentration increases were detected following the application of a commercial elicitor in Red Globe^46^.

Other research reported instead that the stimulation of the production of secondary metabolites in different plant species can be linked to a decrease in the concentration of primary metabolites^47^, and this has also been verified in grapevine leaves^48^ and berries^49^ where the application of different elicitors involved a decrease in sugar concentrations.

In our experiment, a specific inactivated yeast (*Saccharomyces cerevisiae)*, applied on the whole canopy of Sangiovese vines twice during three consecutive seasons quite different in terms of climatic conditions, never affected technological maturity of the berries. Several researches conducted on grapevine using different types of elicitors agree with our findings. In particular, no effects were detected on Syrah^30^, Tempranillo^32^, Merlot, Gaglioppo, Glera and Pinot grigio^50^ treated with yeast derivatives, in Tempranillo treated with methyl jasmonates^32^ and in Cabernet Sauvignon^23^ treated with oligosaccharides deriving from pectin. According to previous results, our data suggest that the application of the yeast derivative on Sangiovese does not affect sugar accumulation, titratable acidity and pH across different climatic seasons, while vintages showed the greatest influence on sugar accumulation^51^.

Several papers have documented the effect of changing climatic conditions on anthocyanin accumulation with particular attention to the increase of diurnal^39,40^ and nocturnal temperatures^52^ on different cultivars. As already demonstrated by several studies^39,40^, the accumulation of anthocyanins in Sangiovese is strictly dependent on temperature trends and prolonged maximum temperatures higher than 35 °C can drastically reduce the accumulation. In our experimental conditions, the application of specific inactivated yeast was able to induce anthocyanin accumulation in two out of the three years in the trial (2016 and 2018). In 2016 temperatures during berry ripening were mostly favourable to anthocyanin accumulation, with maximums that never exceeded 35 °C and they were sometimes lower than 30 °C for several days. In 2018, the weather conditions were different and in general not favourable to anthocyanin accumulation, with several days having maximum temperatures equal to or even higher than 35 °C. Despite these differences, in both years the yeast derivative was able to increase anthocyanin production similarly to what observed in other studies conducted on Sangiovese following the application of a seaweed extract^53^. In our conditions, this effect seems to be stable across different seasons in Sangiovese, under different thermal regimes that could be considered favourable (2016) or unfavourable (2018) to the accumulation of anthocyanins. The 2017 weather conditions were not so different from those in 2018, which was even warmer than 2017 in terms of DD during the season, but the aspect that may be responsible for the different vines response to the treatment was the prolonged and exceptional heat wave immediately after the second application of the specific inactivated yeast, which would have interfered with the action of the elicitor.

It is very interesting that the maximum temperatures recorded during 2017 heat wave (almost 40 °C for 7 consecutive days) were also able to prevent any positive effect of the treatment on the induction of the genes involved in anthocyanin biosynthesis. On these bases, we can affirm that in C and LVM berries the accumulation of anthocyanins proceeded in the same way because the high temperatures contrasted the enhancement, induced by the elicitor, of the expression of the genes involved in anthocyanin biosynthesis.

The mechanism by which an elicitor results in induction of the genes is not yet clear. The perception by the plant of a microbial-derived elicitor, such as a yeast derivative, is due to the presence of yeast structural components like chitin or β-glucans^54^, which can trigger a complex cascade of signalling events, including the production of reactive oxygen species^55^ (ROS). Furthermore, recent researches report that after the application of different elicitors, it can be detected an increase of expression of several defence-related genes or inactivation of non-defence-related genes, a transient phosphorylation/dephosphorylation of proteins and an activation of enzymes involved in the biosynthetic pathways of many secondary metabolites^56^. A recent phylogenetic analysis in grapevine highlighted the presence of three proteins (VvLYK1-1, VvLYK1-2, and VvLYK1-3) that are putative orthologues of the chitosan elicitor receptors of Arabidopsis, AtCERK1 (also known as LYK1), and of rice, OsCERK1^18^. Similarly, an orthologue of AtFLS2, which in Arabidopsis is a receptor of flagellin, the main building protein of eubacterial flagella, was identified in the grapevine genome^57^. After the perception of the elicitor stimulus by specific receptors, the presence of second messengers leads to a transcriptional rearrangement that includes genes involved in the secondary metabolites pathway^58^ via the activation or de novo biosynthesis of transcription factors that in turn regulate the expression of defence genes encoding enzymes involved in the biosynthesis of secondary signals or secondary metabolites^25,59^. In our study, the increase of expression of several genes involved in the early and late steps of anthocyanin biosynthesis was detected. Considering the genes involved in the early steps of the pathway, the application of the yeast derivative caused the up-regulation in at least one sampling date, even if the temperature seems to play a key role in determining their timing of activation. As these genes are also involved in the biosynthesis of other compounds, it would be interesting to understand whether other flavonoid compounds in addition to anthocyanins could be induced by the yeast derivative. For the genes specifically involved in anthocyanin biosynthesis the effect was instead very stable among the seasons, implying the anthocyanin accumulation’s rise. Despite the biosynthetic pathway and the genes involved in anthocyanin biosynthesis are well-known and have been studied in depth, sometimes the expression of the relative genes does not reflect the real accumulation of anthocyanin compounds due to the presence of degradation processes^39,60^ and post-transcriptional or post-translational regulations^61^. Conversely, in our research a strong relation between anthocyanin accumulation and gene expression was detected. In particular, the enhancement of anthocyanin accumulation was detected regardless seasonal conditions, when an increase of UFGT gene expression just 48 h after the treatment and this could be a useful indicator of the treatment effectiveness in increasing anthocyanins.

Based on the results of the research we can affirm that the inactivated yeast is able to induce the expression of the genes involved in anthocyanin biosynthesis and their accumulation. However, in the case of extreme climatic conditions during veraison, when maximum temperatures can be over 40 °C, the seasonal effect could prevail over the positive elicitation effect. Furthermore, this study confirms for Sangiovese the crucial role of climatic conditions during veraison, the most important phase for anthocyanin accumulation, determining their concentration throughout the ripening process.

## Materials and methods

### Plant material and experimental design

The trial was conducted in three subsequent years (2016, 2017 and 2018) on 6 uniform potted plants of six years old *V. vinifera* cv Sangiovese clone 12 T grafted on SO4 rootstock vines trained to Guyot system with two canes and 16–18 buds per plant. Vines were grown outdoor in the facilities of the Department of Agricultural and Food Sciences (DISTAL), the University of Bologna (Italy). The vines, planted in 2010 in 30 L pots filled with a soil mixture (39% sand, 39% silt and 22% clay) with an organic matter content of 1.8% and pH of 7.8, were evened out each year before blooming at 15 clusters. The trial compared untreated control plants (C) and plants treated with LalVigne Mature (LVM), 100% specific inactivated yeast, *Saccharomyces cerevisiae* (Lallemand Inc., Canada). The product was sprayed following the company’s instructions, with a product concentration equal to 1 kg/ha on the same vines during the three years trial. Plants were sprayed till run off twice during ripening: at the beginning of veraison (A1, corresponding to 26/07/16, 19/07/17 and 28/07/18) and when 70% of berries were coloured (A2, corresponding to 03/08/16, 31/07/17 and 04/08/18). Water treated plants were used as control. The vines were adequately irrigated during the season. Mean daily air temperature and rainfall data were recorded each year from April to September at a weather station located close to the trial site.

Samples of berries were taken from C and LVM plants to perform biochemical and molecular analysis just before the first treatment (T0), 48 h after the first treatment, 48 h after the second treatment, one and three weeks later and, finally, at harvest. In particular, for T0 sampling, the molecular analyses were conducted on three different samples, each deriving by the combination of C and LVM berries. Furthermore, in 2017, due to extreme climatic conditions, an extra sampling was conducted a week before harvest for anthocyanin analysis.

At harvest, the crop of each plant was harvested separately and the number of clusters and their weight were recorded.

### Must analyses and anthocyanin separation via HPLC

In all sampling dates and years, 10 berries were collected from each vine for the analysis of °Brix, pH, and titratable acidity. Contemporarily, 20 berries per vine were collected for anthocyanin analysis via HPLC, following the method described in Mattivi et al.^42^ and using a Waters 1525 HPLC (Waters, Milford, MA) equipped with a diode array detector (DAD) and a Phenomenex (Castel Maggiore, BO, Italy) reversed-phase column (RP18, 250 mm × 4 mm, 5 μM). Anthocyanins were quantified at 520 nm using an external calibration curve with malvidin-3-glucoside chloride as the standard (Sigma-Aldrich).

### RNA extraction and gene expression analysis

At each sampling date, except harvest, 20 berries were sampled from each vine and RNA extraction was performed on berry skin using the Spectrum Plant Total RNA kit (Sigma-Aldrich)^50^. RNA quality and quantity were determined using a Nanodrop-1000 spectrophotometer (Thermo Scientific, Wilmington, DE, USA). One microgram of extracted RNA was treated with two units of DNase I (Promega, Madison, USA) and then reverse transcribed using Improm-II Reverse Transcriptase (Promega, Madison, USA), according to the manufacturer’s instructions. Real Time quantitative PCR analysis was performed with a dilution of cDNA (1:20) to which a master mix containing SYBR Green (Applied Biosystems, Foster City, CA, USA) and the primers of the genes of interest were added. The PCR reaction was conducted on an ABI PRISM Step One Plus system (Applied Biosystems, Foster City, CA, USA), as reported in Pastore et al.^40^. Non-specific PCR products were identified by the dissociation curves. Each reaction was performed in 3 technical replicates, using actin^39^ and ubiquitin^58^ as housekeeping genes. The expression of all the genes involved in the first and late steps of anthocyanin biosynthesis were assessed and primers retrieved from the literature: PAL1 (phenylalanine ammonia lyase) in Belhadj et al.^63^; CHS1, CHS2, CHS3 (chalcone synthase 1, 2, 3), DFR (dihydroflavonol reductase), LDOX (leucoanthocyanidin dioxygenase) and UFGT (UDP-glucose:flavonoid 3-O-glucosyl transferase) from Goto-Yamamoto et al.^64^; CHI1, CHI2 (chalcone isomerase 1, 2), F3H1, F3H2 (flavanone 3-hydroxylase 1,2) and MYBA1 from Jeong et al.^65^; GST4 (glutathione S-transferase) from Conn et al.^43^. In the case of PAL, which is in different isoforms in the grapevine genome, the gene encoding the isoform PAL1 was chosen, as from the literature it resulted presumably involved in the response to elicitors in grape^63^. Amplification efficiency was calculated with the LinRegPCR software and used in the calculation of the MNE (Mean Normalized Expression), as reported in Simon^66^. The mean normalized expression (MNE)-value was calculated for each sample referred to the actin and ubiquitin expressions according to the Simon equation^66^.

### Statistical analyses

Technological ripening and anthocyanin accumulation data were subjected to a combined analysis of variance over years using the mixed procedure available in SAS v9.0 (SAS Institute, Inc.). For gene expression data one-way analysis of variance (ANOVA) was conducted.

Treatment comparisons were analysed using the Tukey’s test for pairwise comparison with mean separation by P < 0.05.

## Supplementary information

Supplementary information
