# Supplementary material for: Foliar application of specific yeast derivative enhances anthocyanins accumulation and gene expression in Sangiovese cv (Vitis vinifera L.)
Source: Sci Rep. 2020 Jul 15;10:11627. doi: 10.1038/s41598-020-68479-0 (PMC7363895; doi:10.1038/s41598-020-68479-0)

Foliar application of specific yeast derivative enhances anthocyanins accumulation and gene expression in Sangiovese cv (*Vitis vinifera* L.).

C. Pastore[1], G. Allegro[1], G. Valentini[1], A. Pizziolo[1], F. Battista[2], F. Spinelli[1], I. Filippetti[1*]
[1]Department of Agricultural and Food Sciences, University of Bologna, Viale Fanin, 44, 40127, Bologna, Italy.
[2]Lallemand Italy, Via Rossini 14/B, 37060 Castel d'Azzano, Verona, Italy.
*Corresponding author: ilaria.filippetti@unibo.it

Supporting Information

Supplementary Table 1. Anthocyanin profile (%) of the five glucosylated and acylated anthocyanins in control (C) and yeast derivative (LVM) treated berries at harvest in 2016, 2017 and 2018.

		Delphinidin-3-glucoside (%)	Cyanidin-3-glucoside (%)	Petunidin-3-glucoside (%)	Peonidin-3-glucoside (%)	Malvidin-3-glucoside (%)	Acylated forms (%)
2016	C	14.47	27.61	14.17	13.07	28.64	2.04
	LVM	12.43	30.98	12.43	17.22	25.97	2.75
	sign.	ns	ns	ns	ns	ns	ns
2017	C	14.66	37.22	12.48	15.58	18.07	1.99
	LVM	16.62	39.87	12.77	14.17	15.39	1.21
	sign.	ns	ns	ns	ns	ns	ns
2018	C	11.88	23.08	13.60	17.73	32.53	1.18
	LVM	11.44	20.91	13.42	17.70	35.28	1.25
	sign.	ns	ns	ns	ns	ns	ns

Different letters indicate statistical significance ($P < 0.05$) between the treatments according to Tukey test.

Supplementary Figure 1. Soluble solids concentration in control (C) and yeast derivative (LVM) treated berries during 2016 (A), 2017 (B) and 2018 (C) seasons. Arrows indicate the date on which first (A1) and second (A2) treatments were applied for each year. Error bars indicate standard error (n = 3).

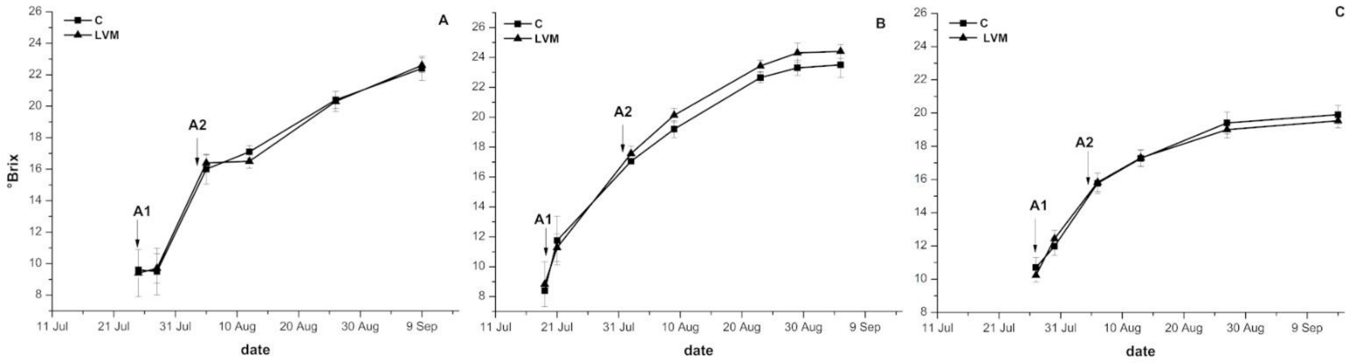

Supplementary Figure 2. Titratable acidity in control (C) and yeast derivative (LVM) treated berries during 2016 (A), 2017 (B) and 2018 (C) seasons. Arrows indicate the date on which first (A1) and second (A2) treatments were applied for each year. Error bars indicate standard error (n = 3).

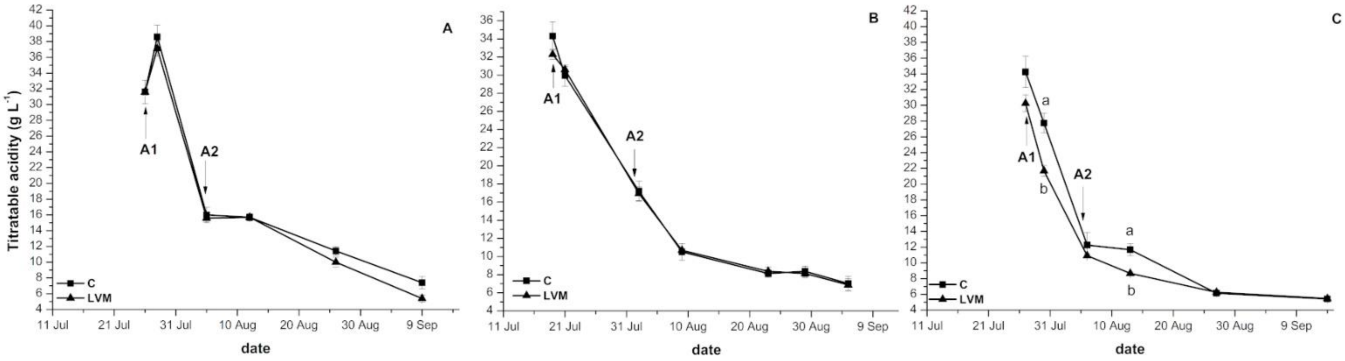

Supplementary Fig. 3. Expression analyses of genes on 2017 samples in control (C) and yeast derivative (LVM) treated berries: PAL1 (A), CHS1 (B), CHS2 (C), CHS 3 (D), CHI 1 (E), CHI 2 (F), F3H1 (G) and F3H2 (H). Arrows indicate the date on which first (A1) and second (A2) treatments were applied for each year. T0 analyses were conducted on three different samples, each deriving by the combination of C and LVM berries. Error bars indicate standard error (n = 3). Means followed by different letters differ significantly, as calculated by Tukey statistical analysis (P<0.05).

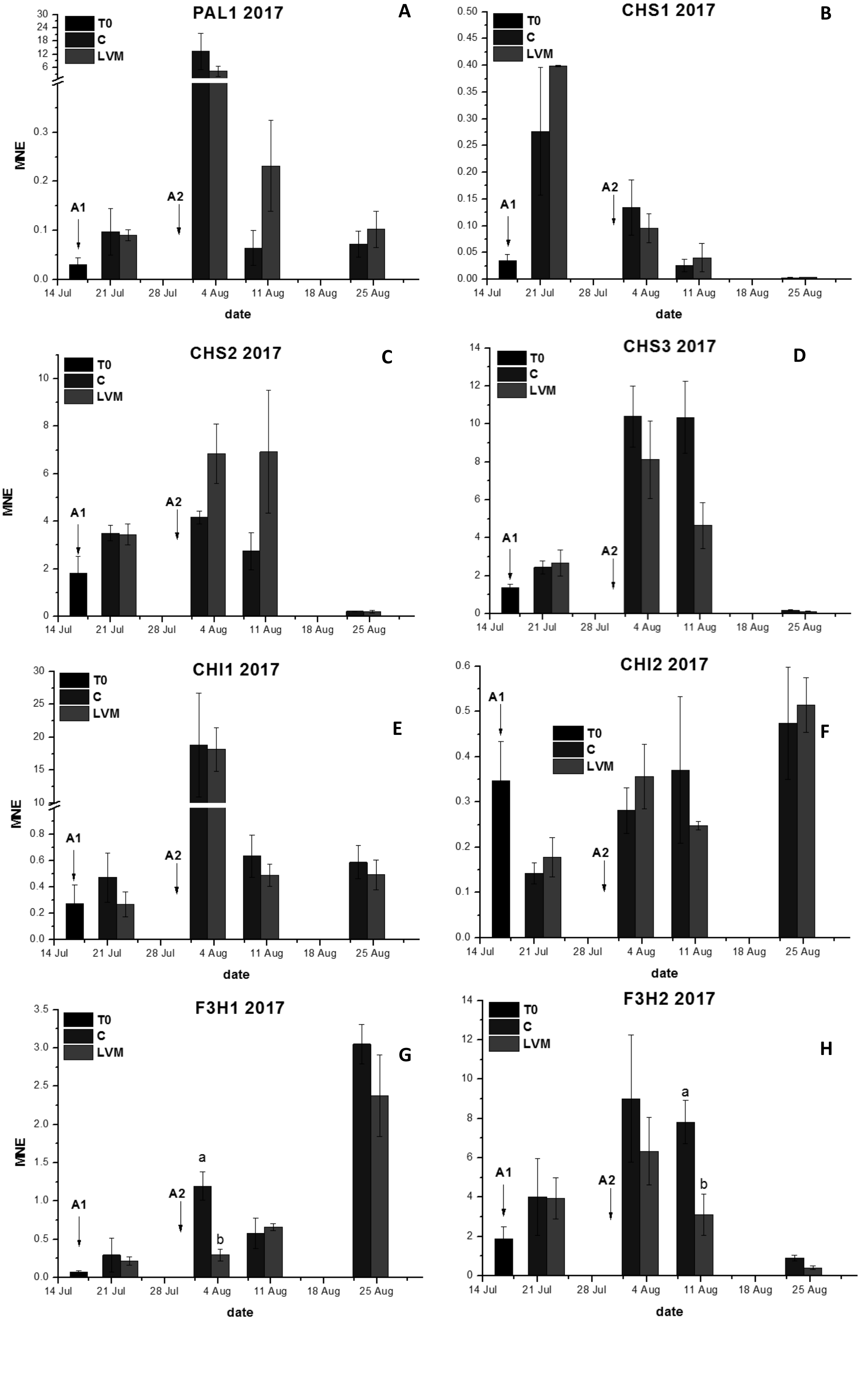

Supplementary Fig. 4. Expression analyses of genes involved in the late steps of anthocyanin biosynthesis and in their regulation and transport on 2017 samples in control (C) and yeast derivative (LVM) treated berries: DFR (A), LDOX (B), UFGT (C), MYBA1 (D), GST4 (E). Arrows indicate the date on which first (A1) and second (A2) treatments were applied for each year. T0 analyses were conducted on three different samples, each deriving by the combination of C and LVM berries. Error bars indicate standard error (n = 3). Means followed by different letters differ significantly, as calculated by Tukey statistical analysis (P<0.05).

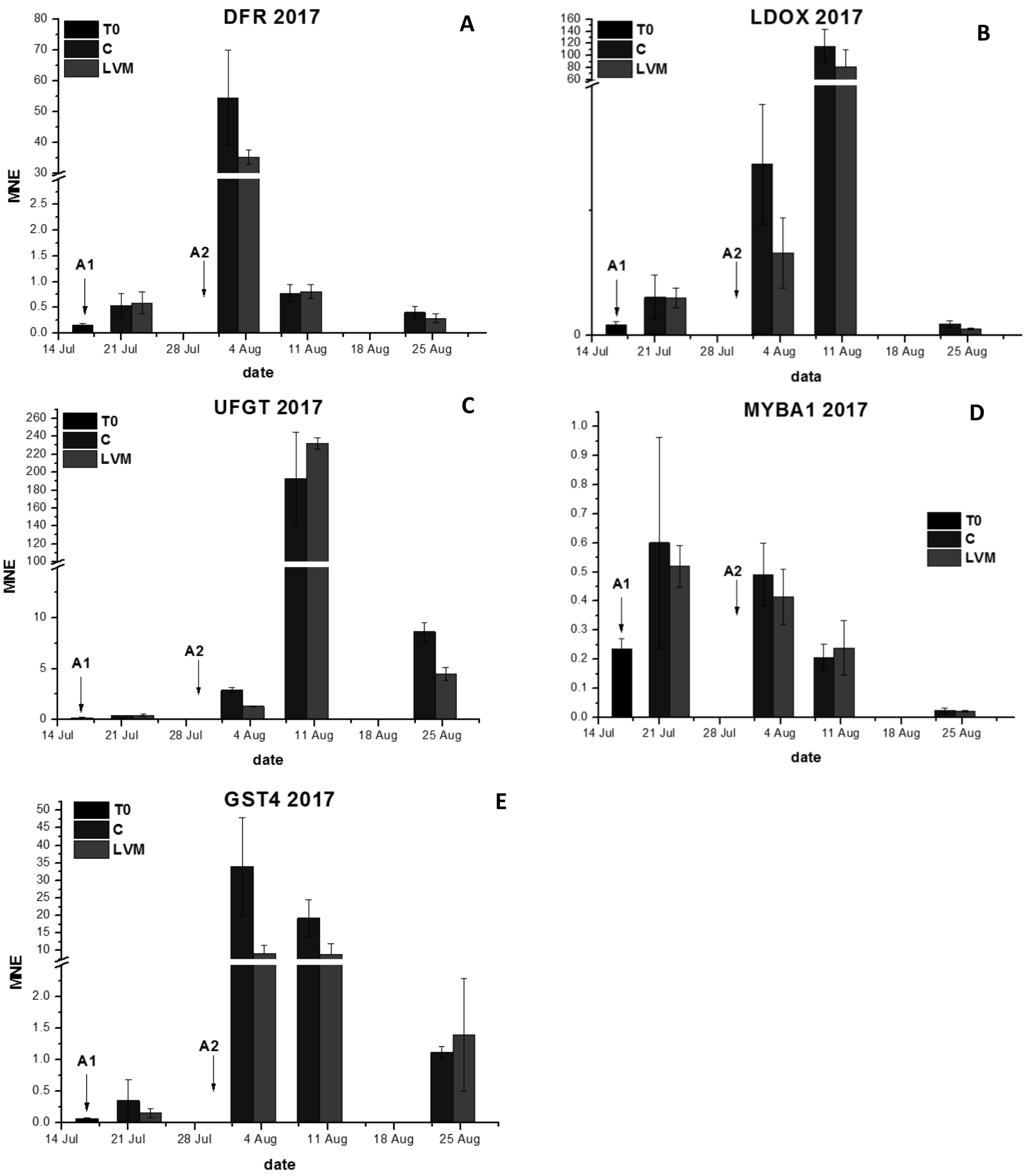

Supplement: Supplementary file 1 — Supplementary information [file 41598_2020_68479_MOESM1_ESM.pdf]
